# β-conglycinin combined with fenofibrate or rosuvastatin have exerted distinct hypocholesterolemic effects in rats

**DOI:** 10.1186/1476-511X-11-11

**Published:** 2012-01-13

**Authors:** Ederlan S Ferreira, Maraiza A Silva, Aureluce Demonte, Valdir A Neves

**Affiliations:** 1 Department of Food and Nutrition, School of Pharmaceutical Sciences, São Paulo State University -UNESP, Araraquara, SP, Brazil

**Keywords:** β-conglycinin, cholesterol-lowering drugs, hypercholesterolemic diet, rats.

## Abstract

**Background:**

There is increasing interest in non-pharmacological control of cholesterol and triglyceride levels in the plasma and diet-drug association represent an important area of studies. The objective of this study was to observe the hypocholesterolemic effect of soybean β-conglycinin (7S protein) alone and combined with fenofibrate and rosuvastatin, two hypolipidemic drugs.

**Methods:**

The protein and drugs were administered orally once a day to rats and the effects were evaluated after 28 days. Wistar rats were divided into six groups (n = 9): hypercholesterolemic diet (HC), HC+7S protein (300 mg.kg-1 day-1) (HC-7S), HC+fenofibrate (30 mg.kg-1 day-1)(HC-FF), HC+rosuvastatin (10 mg.kg-1 day-1)(HC-RO), HC+7S+fenofibrate (HC-7S-FF) and HC+7S+rosuvastatin (HC-7S-RO).

**Results:**

Animals in HC-7S, HC-FF and HC-RO exhibited reductions of 22.9, 35.8 and 18.8% in total plasma cholesterol, respectively. In HC-7S-FF, animals did not show significant alteration of the level in HC+FF while the group HC-7S-RO showed a negative effect in comparison with groups taking only protein (HC-7S) or drug (HC-RO). The administration of the protein, fenofibrate and rosuvastatin alone caused increases in the plasma HDL-C of the animals, while the protein-drug combinations led to an increase compared to HC-FF and HC-RO. The plasma concentration of triacylgycerides was significantly reduced in the groups without association, while HC-7S-FF showed no alteration and HC-7S-RO a little reduction.

**Conclusion:**

The results of our study indicate that conglycinin has effects comparable to fenofibrate and rosuvastatin on the control of plasma cholesterol, HDL-C and triacylglycerides, when given to hypercholesterolemic rats, and suggests that the association of this protein with rosuvastatin alters the action of drug in the homeostasis of cholesterol.

## Background

Some proteins and peptides have been under study on account of their important biological function in relation to lipid metabolism [1-3]. Research has demonstrated the importance of leguminous seeds, as a component of the diet including their beneficial effect on the control of cholesterol and triacylglycerols in the blood, with an emphasis on their proteins and in particular soybean proteins [4,5]. *In vitro *and *in vivo *studies have shown that ingestion of protein isolate and/or protein fractions of soybean, as the sole source of protein or even a daily dose, improves the control of plasma cholesterol and triglycerides [6-15]. A recent analysis of various studies about the effects of soybean in the diet on the circulation levels of cholesterol indicates a variable reduction in LDL-C (from 7.9 to 10.3%) attributed to intrinsic and extrinsic effects of the soy protein foods [16]. Although these studies in various models have achieved progress in this area, the mechanism of the reduction of cholesterol and triacylglycerides in the serum by soybean proteins are still not clear. Nevertheless, the observed effects of soybean proteins was the basis on which the US Food and Drug Administration (FDA) made a recommendation that 25 g of soybean protein be ingested to decrease total cholesterol and the LDL-cholesterol fraction [17]. A lot of studies have stressed the diet as an important form of intervention, alone or together with drugs, in the context of hyperlipidemia or hypercholesterolemia in their various forms [4,18]. The treatment of hyperlipidemia and hypercholesterolemia with a diet-drug combination is well recognized and has been growing in importance in recent times [19]; nevertheless, these combination therapies have received less attention than multi-drug therapy [18,20]. Fibrates and their derivatives are hypolipidemic agents widely used in the treatment of combined hyperlipidemias, however they are particularly effective at reducing serum triacylglycerols and improving serum high-density lipoprotein (HDL) [21,22].

Statins are used in the treatment of hypercholesterolemia, and the action of this class of drugs depends on their generation and dose. These drugs have an inhibitory action on cholesterol synthesis via the mevalonate pathway [18,23]. The positive effects of β-conglycinin soybean protein, taken as a daily dose or a sole protein source by rats fed a hypercholesterolemic diet, on TC and TG levels have been described in previous studies [13-15]. However, the combination of this protein with other antihyperlipidemic drugs has not been much reported. Drug-diet approaches may help in controlling the prescription of drugs, but studies are needed to be performed to explore novel combinations and assess the potential safety and effectiveness of the association, comparing its efficacy with treatment by each component alone. To observe of the effect of soybean β-conglycinin alone and in combination with two drugs effective in the treatment of dyslipidemia, fenofibrate and rosuvastatin, the present study was conducted to determine whether the association of β-conglycinin soy protein and either drug could result in an alteration of the action of the protein or the drug alone.

## Methods

### Isolation and characterization of β-conglycinin (7S)

Soybean flour from the local market (60 mesh) was defatted with hexane (1:8 w/v flour to solvent ratio) by rocking at room temperature for 12 h, followed by reextraction in the same solvent (1:6 w/v), dried in air at room temperature and used for protein extraction. Soybean β-conglycinin was isolated by the method of Nagano et al. [24] with some modifications. Defatted soybean flour was extracted by shaking with water (1:15 w/v flour to water ratio) adjusted to pH 7.5. The soluble extract was centrifuged at 2000 × *g *for 30 min. The supernatant was treated with anhydrous sodium bisulphite (0.98 g/L) and the pH adjusted to 6.4. The solution was stirred and stored on ice overnight, followed by centrifugation at 6500 × *g *for 20 min. The insoluble fraction, containing 11S globulin, was washed, centrifuged again under the same conditions, suspended in distilled water, dialyzed overnight against distilled water in appropriate dialysis sacs (pore size of about 10 kDa) and lyophilized. The soluble fraction was treated with 0.25 mol/L NaCl and adjusted to pH 5.0 and, after 1 h, centrifuged at 9000 × *g *for 30 min. The precipitate (7S or β-conglycinin) was solubilized in 0.25 mol/L sodium phosphate buffer (pH 7.0) and stored at -14°C. All steps were realized at 4°C. All chemicals were of analytical purity. Protein was determined by the method of Lowry et al. [25], using bovine serum albumin (BSA) as a standard. Electrophoresis of 7S-globulin was performed on 10 g/100 g polyacrylamide gels containing 0.1% sodium dodecyl sulfate (SDS), as described by Laemmli [26], in a Mini Protean II cell (Bio-Rad, Hercules, CA, USA)(SDS-PAGE). The gels were stained in Coomassie Brilliant Blue (R-250) and destained by diffusion in methanol-acetic acid-water (1:1:8, v/v/v). Marker proteins of known molecular weight (MW) were rabbit muscle phosphorylase b, bovine serum albumin, hen egg white albumin, bovine carbonic anhydrase, soybean trypsin inhibitor and hen egg white lysozyme. Gel images were digitalized and analyzed by densitometry with an alpha Imager 6.0 scanner (Alpha Innotech^®^, San Leandro, CA, USA), to quantify the bands and level of purity (data not shown). The content and homogeneity of the isolated β-conglycinin and glycinin, determined by SDS-PAGE and densitometric gel analysis, showed that these were the main fractions. The gels showed the characteristic bands for subunits α, α' and β of conglycinin (7S). The procedure used to isolate 7S did not cause changes in the subunits; this step was essential to ensure a pure protein, suitable for the *in vivo *studies [15].

### Animals, diets and experimental design

This study was performed with the approval of the Committee on Animal Research of the University (UNESP) and these experiments were approved by the Research Ethics Committee of the School of Pharmaceutical Sciences, São Paulo State University (UNESP) at Araraquara (res. 19/2007). All procedures were performed in accordance with the principles in the Guide for the Care and Use of Laboratory Animals (National Research Council, 1985). Fifty-four male weanling Wistar rats, weighing 148 ± 12.3 g were obtained from the Central Laboratory for animals of UNESP at Botucatu. The animals were acclimatized for a week and fed standard laboratory rat chow (Purina^®^). After this period, they were randomly divided into 6 hypercholesterolemic groups (HC) (*n = 9*): (1) control HC; (2) 7S globulin, 300 mg.kg^-1 ^day^-1 ^(HC+7S); (3) fenofibrate group (HC+FF, 30 mg.kg^-1 ^day^-1^); (4) rosusvatatin group (HC+RO 10 mg.kg^-1 ^day^-1^); (5) β-conglycinin/fenofibrate group (HC+7S+FF, 300 and 30 mg.kg^-1 ^day^-1^) and (6) β-conglycinin/rosuvastatin group (HC+7S+RO, 300 and 10 mg.kg^-1 ^day^-1^). All HC groups were fed an AIN-93 diet, following the recommendation of American Institute Nutrition 93-Maintenance [27], modified by adding 10 g.kg^-1 ^cholesterol and 5 g.kg^-1 ^g cholic acid [13,28], as described in Table 1. Animals were individually housed in stainless steel cages, with free access to food and water. The room temperature was maintained at a constant 23 ± 2°C and relative humidity at 60 ± 5%, with a 12-12 h light:dark cycle, lights on at 6:00 a.m. The proteins (7S globulins) and drugs were administered daily by gavage at 9:00 and 14:00 hours, in proportion to the body weight of each animal, while vehicle alone was administered to HC groups. Body weight, food intake, fecal excretion and feeding efficiency were measured every other day for comparative analysis between groups during the 28 days of the experiment. The feeding efficiency coefficient was calculated as the ratio of weight gain/daily intake × 100.

**Table 1:** Composition (g/kg diet) of the hypercholesterolemic diet and treatments by gavage*

Ingredient	Diets					
	
	HC	HC+7S	HC+FF	HC+RO	HC+7S+FF	HC+7S+RO
Casein^1^	150	150	150	150	150	150
Starch	590.57	590.87	590.87	590.87	590.87	590.87
Sucrose	100	100	100	100	100	100
Soybean oil	40	40	40	40	40	40
Cellulose powder	50	50	50	50	50	50
Mineral mixture^2^	35	35	35	35	35	35
Vitamin mixture^2^	10	10	10	10	10	10
L-Cystine	1.8	1.8	1.8	1.8	1.8	1.8
Choline birtatrate	2.5	2.5	2.5	2.5	2.5	2.5
Cholesterol^3^	1	1	1	1	1	1
Cholic acid^1^	5	5	5	5	5	5
*Treatment *(mg/kg/day)						
β-conglycinin	-	300.0	-	-	300	300
Fenofibrate	-	-	30	-	30	-
Rosuvastatin	-	-	-	10	-	10

*HC = hypercholesterolemic diet (AIN-93 composition + 1% cholesterol and 0.5% cholic acid); HC+7S = HC diet + β-conglycinin 300 mg/Kg/day; HC+FF = HC diet + fenofibrate 30 mg/Kg/day; HC+RO = HC diet + rosuvastatin 10 mg/kg/day; HC+7S+FF = HC diet + β-conglycinin + fenofibrate 300 and 30 mg/kg/day and HC+7S+RO = HC diet + β-conglycinin + rosuvastatin 300 and 10 mg/kg/day. ^1^Sigma-Aldrich, Co., USA, ^2^PragSoluções^®^, Co., Brazil. ^3^Reagen, Co., USA.

### Blood collection, organ weight and tissue collection

On the last day, the animals were deprived of food for 12 h and euthanized by guillotine. Blood was then collected in tubes containing gel separator SST II (Vacutainer BD D^®^, Franklin Lakes, NJ, USA) and centrifuged at 1900 × *g *for 15 min. The serum was separated, stored at -24°C and used for biochemical analysis. Retroperitoneal fat, liver and heart were removed, washed immediately in cold saline, dried and weighed, frozen and stored at -40°C for a period of less than a month for subsequent comparative analysis.

### Biochemical analysis in plasma and liver

Plasma total cholesterol (TC) of all groups was measured by the liquid cholesterol CHOD-PAP method described by Stockbridge et al. [29]. Serum high density lipoprotein (HDL-C) was measured by the HDL cholesterol precipitant method, as described by Assmann [30]. Triglycerides (TG) were measured by the liquid triglyceride GPO-PAP method, as described by Annoni et al. [31]. All analyses were carried out by enzymatic colorimetric methods, using commercially available reagent kits (Laborlab^®^Co., Ribeirão Preto, Brazil). The non-HDL-cholesterol fraction (non-HDL-C or LDL-C + VLDL-C) was calculated as the difference between the TC and HDL-C. The atherogenic index (AI) (TC-HDL-C/HDL-C) was calculated, as proposed by Liu et al. [32], and compared among groups. Hepatosomatic index (HIS) was calculated as follows: liver weight/body weight × 100. Liver lipids were extracted by the method of Haug et al. [33]. The liver TC and TG concentrations were measured as described earlier for plasma analysis.

### Statistical Analysis

Data were analyzed by one-way analysis of variance (ANOVA) and *Bonferroni t-test *multiple comparisons versus the control group and multiple range tests, employing SigmaStat^® ^3.5 software (Dundas software, Germany, 1999). All values are presented as mean ± standard error for nine values per group. A difference was considered statistically significant when *P *< 0.05.

## Results

### Isolation of the soybean β-conglycinin

SDS-PAGE electrophoresis, conducted on the 7S globulin after separation from other protein components in soybean flour, showed the characteristic bands of conglycinin (7S), arising from α, α', β polypeptide subunits, and densitometric gel analysis showed that this was the main fraction, containing 92.2% of the protein. The isolation procedure did not cause any change in the subunits and was important to obtain purified protein suitable for the *in vivo *studies [14,15]. Our previous results and those decribed by Duranti et al. [13] have shown that the addition of 1% cholesterol and 0.5% cholic acid to the AIN-diet is sufficient to induce hypercholesterolemia and hypertriglyceridemia in rats. The mechanism of these effects on the lipid metabolism of rats was reported by Wang et al. [34], recently.

β-conglycinin protein and drugs were given by gavage in single daily doses in the experimental design as described in methods. Table 2 shows the effects of daily administration of β-conglycinin, fenofibrate and rosuvastatin alone and in drug-protein combinations, on the food consumption, weight gain, feeding efficiency ratio and fecal excretion of rats fed the cholesterol-enriched diets. After 28 days, no statistical differences (*p >*0.05) were found in body weight gain between groups during the experimental period, nor any significant (*p *> 0.05) alterations in the feeding efficiency ratio or food intake between groups (Table 2). However, the animal fecal excretion of the HC group was higher by 18.1, 18.1, 42.9, 21.7 and 50.7% than groups HC+7S, HC+FF, HC+RO, HC+7S+FF and HC+7S+RO, respectively (*p *< 0.001), despite the lack of differences in food intake or feeding efficiency ratio between groups. However, the rosuvastatin-treated animals did show an intake 8.72% higher than HC animals (*p *< 0.001). The fenofibrate and rosuvastatin groups showed increases of 55.8 and 63.2% (*p *< 0.001), and 26.1 and 21.1% (*p *< 0.05), in the relative liver weight and in the hepatosomatic index (HIS), respectively (Table 3). The simultaneously administration of protein and drugs reduced these effects by 8.4 and 16.1% for fenofibrate (HC+7S+FF) and 24.8 and 16.7% for rosuvastatin (HC+7S+RO), relative to groups HC+FF and HC+RO, respectively. Alterations in heart weight and the ratio heart weight/body weight were not observed.

**Table 2:** Body weight change, food intake, and feeding efficiency ratio (FER) of rats fed HC diets and treated with separate and combined doses of β-conglycinin, fenofibrate and rosuvastatin.

	**Dietary groups**^***1***^					
	
	HC	HC+7S	HC+FF	HC+RO	HC+7S+FF	HC+7S+RO
Initial weight (g/rat)	196.55 ± 4.63	195.26 ± 3.64	193.26 ± 3.64	193.33 ± 2.27	193.18 ± 3.62	194.22 ± 3.14
Final weight (g/rat)	295.01 ± 5.43	300.64 ± 7.71	281.14 ± 4.25	306.11 ± 3.79	306.64 ± 7.71	284.33 ± 8.01
Weight gain (g/rat/day)	3.51 ± 0.32	3.76 ± 0.29	3.14 ± 0.29	4.02 ± 0.16	4.05 ± 0.17	3.22 ± 0.28
Food intake (g/rat/day)	16.74 ± 0.33	16.25 ± 0.22	16.25 ± 0.22	18.20 ± 0.17**	15.61 ± 0.30	17.68 ± 0.25
Fecal excretion (g/rat/day)	3.59 ± 0.12	2.94 ± 0.09*	2.94 ± 0.09*	2.05 ± 0.12**	2.83 ± 0.09*	1.77 ± 0.13**
Feeding efficiency (%)	21.01 ± 1.44	23.05 ± 1.93	23.21 ± 1.93	21.35 ± 0.85	20.90 ± 1.51	18.25 ± 1.34

^1^Values are means ± SEM for nine rats. *p < 0.05 and **p < 0.001 by the Bonferroni t-test for multiple comparisons versus control group (HC). HC = hypercholesterolemic diet; HC+7S = HC diet + β-conglycinin 300 mg/kg/day; HC+FF = HC diet + fenofibrate 30 mg/kg/day; HC+RO = HC diet + rosuvastatin 10 mg/Kg/day; HC+7S+FF = HC diet + β-conglycinin + fenofibrate, 300 and 30 mg/kg/day respectively, and HC+7S+RO = HC diet + β-conglycinin + rosuvastatin, 300 and 10 mg/kg/day respectively.

**Table 3:** Tissue weight of rats fed HC diets and treated with doses of β-conglycinin, fenofibrate, rosuvastatin and mixtures

	**Dietary groups**^***1***^					
	
	HC	HC+7S	HC+FF	HC+RO	HC+7S+FF	HC+7S+RO
Liver weight (g/rat)	13.54 ± 0.36	13.55 ± 0.87	21.10 ± 0.69**	17.07 ± 0.92*	19.32 ± 0.38**	12.83 ± 0.51
Heart (g/rat)	1.03 ± 0.04	0.94 ± 0.03	0.95 ± 0.03	1.26 ± 0.05	0.96 ± 0.02	0.89 ± 0.05
Hepatossomatic índex* (HI)	4.59 ± 0.14	4.41 ± 0.20	7.51 ± 0.27**	5.56 ± 0.27*	6.30 ± 0.12**	4.63 ± 0.16
Heart weight/body weight (g/100 g)	0.34 ± 0.01	0.30 ± 0.02	0.34 ± 0.01	0.41 ± 0.02*	0.33 ± 0.01	0.32 ± 0.01

^1^Values are means ± SEM for nine rats. *p < 0.05 and **p < 0.001 by the Bonferroni t-test multiple comparisons versus control group (HC). HC = hypercholesterolemic diet; HC+7S = HC diet + β-conglycinin 300 mg/kg/day; HC+FF = HC diet + fenofibrate 30 mg/kg/day; HC+RO = HC diet + rosuvastatin 10 mg/kg/day; HC+7S+FF = HC diet + β-conglycinin + fenofibrate, 300 and 30 mg/kg/day respectively, and HC+7S+RO = HC diet + β-conglycinin + rosuvastatin, 300 and 10 mg/kg/day respectively. Hepatossomatic índex (HI) = liver weight/body weight × 100.

### Effects on plasma lipids

The levels of plasma lipids in group HC were significantly different (*p *< 0.001) from those fed a standard diet [14]; these animals showed a 43.6% reduction in HDL-C levels and an increase in non-HDL-C and in the atherogenic index of 4 and 7 times, relative to a group on the standard diet in the first part of this study [15]. Relative to HC groups HC+7S, HC+ FF and HC+RO had their total plasma cholesterol (TC) reduced by 22.9 (*p *< 0.01), 35.8 (*p *< 0.001) and 18.8% (*p *< 0.05), respectively (Table 4). There was no significant alteration in the animals that received the 7S protein plus fenofibrate (HC+7S+FF), whereas the association of protein plus rosuvastatin (HC+7S+RO) caused a negative effect, when the TC levels of these groups were compared with those administered only protein (HC+7S) or drug (HC+FF and HC+RO). For the fraction non-HDL-C, a similar effect was observed with reductions of 33.2, 53.1 and 28% (*p *< 0.001) for groups HC+7S, HC+FF and HC+RO, respectively, while decreases of 59.5 (*p *< 0.001) and 15.2% (*p *< 0.05) were registered for groups HC+7S+FF and HC+7S+RO. In contrast to rosuvastatin, the protein-fenofibrate association led to an increase of 11.2% (p < 0.05) (Table 4). The administration of the protein, fenofibrate and rosuvastatin alone caused increases in the plasma HDL-C of the animals by 55.6, 95.6 e 51.1% in relation to HC, respectively, (*p *< 0.001), while the protein-drug associations led to increases of 6 and 37%, compared to HC+FF and HC+RO groups, respectively (*p *> 0.05) (Table 4). The atherogenic index (AI) showed decreases of 56.5, 75.8 and 49.4% in HC+7S, HC+FF and HC+RO (*p *< 0.001) in relation to HC, respectively, as a consequence of these effects. The combination of the protein-FF and protein-RO led to increases in AI of 5.3 and 5.2%, respectively (*p *> 0.05).

**Table 4:** Effect of diets on plasma lipid profiles and hepatic lipid contents in rats fed hypercholesterolemic diets and treated with doses of β-conglycinin, fenofibrate, rosuvastatin and mixtures.

	Dietary groups					
	
	HC	HC+7S	HC+FF	HC+RO	HC+7S+FF	HC+7S+RO
*Plasma (mmol/L)*						
Total cholesterol	3.88 ± 0.22	2.99 ± 0.10*	2.49 ± 0.15**	3.15 ± 0.12*	2.33 ± 0.22**	4.00 ± 0.12
HDL-C	0.45 ± 0.02	0.70 ± 0.05*	0.88 ± 0.13*	0.68 ± 0.06	0.93 ± 0.10**	0.90 ± 0.15*
non-HDL-C	3.43 ± 0.12	2.29 ± 0.10**	1.61 ± 0.06**	2.47 ± 0.14**	1.39 ± 0.12**	2.91 ± 0.22*
Triacylglycerides	0.92 ± 0.05	0.60 ± 0.04**	0.50 ± 0.02**	0.85 ± 0.07	0.49 ± 0.03**	0.70 ± 0.05*
AI*	8.09 ± 0.07	3.52 ± 0.11**	1.96 ± 0.12**	4.09 ± 0.35**	1.53 ± 0.15**	3.67 ± 0.33**
*Liver (μmol/g)*						
Total cholesterol	58.43 ± 0.85	46.22 ± 0.65**	39.58 ± 0.85**	36.04 ± 2.07**	37.86 ± 0.80**	47.13 ± 2.12**
Triacylglycerides	56.97 ± 2.15	48.52 ± 2.28*	60.06 ± 1.76	41.42 ± 2.46**	47.64 ± 2.38*	46.05 ± 2.59*

^1^Values are means ± SEM for nine rats. *p < 0.05 and **p < 0.001 by the Bonferroni t-test multiple comparisons versus control group (HC). HC = hypercholesterolemic diet; HC+7S = HC diet + β-conglycinin 300 mg/kg/day; HC+FF = HC diet + fenofibrate 30 mg/kg/day; HC+RO = HC diet + rosuvastatin 10 mg/kg/day; HC+7S+FF = HC diet + β-conglycinin + fenofibrate, 300 and 30 mg/kg/day respectively, and HC+7S+RO = HC diet + β-conglycinin + rosuvastatin, 300 and 10 mg/kg/day respectively. * AI = atherogenic index ware calculated as proposed by Liu et al. (2006).

Triacylglycerol (TG) plasma concentrations of the animals in groups HC+7S and HC+FF showed significant reductions (*p *< 0.001) of 34.8 and 45.7%, whereas group HC+RO had only a slight reduction of 7.6% (*p *> 0.05), relative to HC. For protein-drug combinations, the β-conglycinin/fenofibrate group (HC+7S+FF) did not show any change, but the β-conglycinin/rosuvastatin group (HC+7S+RO) showed a reduction of 23.9% in the TG levels (*p *< 0.05) relative to the respective drug-treated groups (HC+FF and HC+RO) (Table 4).

### Effects on hepatic lipids

Table 4 shows that groups HC+7S had reductions of 20.9 and 14.8% in the liver cholesterol and triacylglycerol concentrations, compared to HC, respectively (*p *< 0.001). Groups HC+FF and HC+RO had 32.3 and 38.3% less cholesterol than HC, respectively (Table 4); on the other hand, group HC+RO had a 27.3% reduced liver TG concentration, while HC+FF did not show significant alteration in this parameter (*p *< 0.001).

## Discussion

The effects of fibrates and statins as drugs for the treatment of dyslipidemias are well described in the literature and their mechanisms of action practically established [21,23]. The action of these compounds is associated with transcriptional control of triglycerides metabolism by the activation of the transcription factor PPAR-α (peroxisome proliferator-activated receptor) and consequent induction of the enzymes of β-oxidation of fatty acids in mitochondria. Statins inhibit endogenous cholesterol synthesis by inhibition of 3-hydroxy-3-methyl-glutamyl-CoA reductase, the rate-limiting enzyme in cholesterol synthesis. Depending on which generation of statin is used the various doses lead to reductions of LDL-C (low-density lipoprotein cholesterol) in a range between 20 and 60% [23]. Diet components, such as plant sterols/stanols, soluble fibers, omega-3-fatty acids, niacin and soybean components; have been combined with a variety of drugs, in experiments *in vitro *and *in vivo*, to treat dyslipidemia [18].

In our experiment, the differences in body weight gain between groups were not significant, although the weights of the livers of rats consuming the mixture of drugs and 7S-FF were much higher than those of other groups. The group that received rosuvastatin had a liver weight gain lower than those given fenofibrate. The observed increase in liver weight by administration of fenofibrate is reported in the literature. Yamamoto et al. [22] found such an increase on administering fenofibrate at 0.05% in diet of rats, while, Mancini et al. [35] observed an increase of 30.2% in the liver weight of hyperlipidemic rats after daily administration of 320 mg.kg^-1 ^of fenofibrate for 120 days. Ji et al. [36] observed an increase in the liver weight of the animals fed on a hyperlipidemic diet and treated with atorvastatin at a dose of 30 mg.kg^-1 ^day^-1 ^for 8 weeks; the authors also showed an increase in the hepatosomatic index of these animals. These results are consistent with published reports of liver hypertrophy after fenofibrate administration, due to proliferation of peroxisomes and mitochondria. In our study, the raised hepatosomatic index of the animals that received fenofibrate thus resulted from the high liver weight and the insignificant body weight gains of the animals (Table 2 and 3). The protein-drug combination caused reductions of 16 and 16.7% in the hepatosomatic indices of the animals that ingested fenofibrate and rosuvastatin, respectively, and in the latter case practically annulled the effect of the drug alone. Therefore, the β-conglycinin was able to reverse the increase in the liver weight and hepatosomatic index of the animals that ingested rosuvastatin, but not those of the 7S+FF animals.

The protein β-conglycinin has been studied for its effects on dyslipidemia, both as a single protein added to the diet, and as a daily dose [7,8,10,12-14,37,38], and in both cases distinct effects on lipid metabolism were observed. Studies using animals subjected to a daily dose of β-conglycinin [13-15], and our study, show that the 7S protein has an effect comparable to that of rosuvastatin, but weaker than fenofibrate, in reducing levels of total cholesterol. Surprisingly, the β-conglycinin/rosuvastatin combination raised the total cholesterol to a higher level than in any other group, even the hypercholesterolemic group, while the combination with fenofibrate led to lower values of TC than either separately.

The LDL-C/HDL-C ratio is used as risk factor for coronary heart diseases (CHD), on account of the effect of each fraction on the atherosclerotic process [39]. Thus, the atherogenic index (AI) of the hypercholesterolemic diet group (HC), in this experiment, was 8 times higher than that of rats on the casein standard diet [14], as also observed by other authors [10]. All treatments resulted in reductions in AI of the animals, the combination of 7S protein and fenofibrate (HC+7S+FF group) being the most efficient, followed by fenofibrate (HC+FF group), protein (HC+7S group), protein plus rosuvastatin (HC+7S+RO group) and rosuvastatin (HC+RO group), with falls of 81, 76, 57, 55 and 50%, relative to HC, respectively (Table 4). These data indicate that the protein-fenofibrate and protein-rosuvastatin combinations resulted in lower cardiovascular risks than did the drugs alone (Table 4). Even though the combination of protein with rosuvastatin rose the level of total cholesterol approximately to that observed in the group HC, the combination reduced the cardiovascular risk by increasing the HDL-C fraction (Table 4).

The mechanisms of action of these drugs are properly established, as mentioned above. However, the mechanisms of action of soybean proteins on lipid metabolism are not yet clear, as various studies point to different actions, such as increasing the amount of LDL-receptors [7,8,40,41], inhibiting HMG-CoA reductase [42], sequestering bile acids [38], activiting β-oxidation related enzymes [38,43], gene expression [40,43] and others [11,12,38,41]. However, a lot of evidence suggests that the effects of soybean proteins could be due to biologically active peptides produced by digestion of the proteins in the gastrointestinal tract, and that these peptides would be the main agents affecting cholesterol and triacylglyceride metabolism, since the protein itself cannot be absorbed intact.

The behaviors of these combinations suggest a possible synergy between the protein and fenofibrate and an antagonistic effect with rosuvastatin, in relation to serum total cholesterol. Although, in the case of rosuvastatin, the increase in the HDL-C fraction to a level above that with the protein or drug alone may be due to a higher rate of cholesterol catabolism, an important function of this fraction is as a carrier of cholesterol to the liver. Fenofibrate has been characterized as a drug that causes an increase in HDL-C levels [44], while the literature indicates only a slight modification of this fraction by the statins, unrelated to dose [20]. These observations were confirmed by our experiment, but β-conglycinin had a positive effect in combination with rosuvastatin, resulting in a higher increase than with the drug alone. The non-HDL-C levels followed the same trend as total cholesterol. The combinations of protein with the drugs did not affect the TG levels, although the rosuvastatin improved the action of the protein in the combination and reduced the level compared to the drug alone.

The liver cholesterol concentration was reduced by all treatments, relative to HC, the greatest reduction (37%) being seen in the HC+RO group. Nevertheless, 7S-rosuvastatin combination annulled the effect of the drug, showing the same value as the protein alone and much higher than with the drug alone. The 7S-fenofibrate combination resulted in a fall in concentration of hepatic triacylglycerides, relative to HC and to the drug alone (which led to a rise in this level), to the same concentration as HC+FF. However, the fenofibrate, without or with 7S protein, maintained the hepatosomatic index above that of the HC group, due to the appreciable increase in the liver weight of the animals.

The meta-analysis of studies by Anderson et al. [45] demonstrated a 12.5% reduction in the levels of LDL-C for 1.5 to 2 oz of soy protein daily (50 g/d), whereas a recent meta-analysis reported only 4-6% [46]. More recently, Jenkins et al. [16] discussed the effect of cholesterol reduction by soy proteins in light of a review of claims for heart health by the U.S. FDA and a meta-analysis of studies on the action of soy protein; they concluded that soy remains one of the few food components that reduce serum cholesterol when added to the diet. Results in the literature show that soybean protein, when used as the only protein source in the diet affects these serum parameters in various ways, depending on the experimental model, animal, dose and other factors [3]. However, when administered as a daily dose in hypercholesterolemic animals fed a standard casein diet, enriched by cholesterol and cholic acid, it had the effect of reducing TC, LDL-C and TG levels in a dose-dependent manner [13,14]. It is important to note that in our study the β-conglycinin was administered by gavage, alone and/or in combination with the drugs at a concentration equivalent to 2.75-2.90% of the daily total protein intake, and that the doses were given separately to the animals, with a difference of 6 hours between drug and the protein. The fact that the β-conglycinin was isolated may be the cause of its higher effect on the cholesterol metabolism relative to the total protein or soybean protein products. We may note that, despite the use of various experimental models with different *in vitro *and *in vivo *approaches, the mechanism of action of soy protein on lipid metabolism is still controversial and deserves further study, especially in relation to its interactions with drugs of similar effect. At the moment, studies to reveal the mechanism of action of β-conglycinin, alone and combined with statins are in progress in our lab.

## Conclusion

The 7S soybean protein-fenofibrate combination was shown to have a positive in reducing the serum total cholesterol levels of hypercholesterolemic animals, as well as raising the levels of HDL-C, and relative to the protein and drug alone. This enhancing effect was reflected in a greater reduction of the cardiovascular risk by up to 81% relative to HC animals. This combination also had a positive effect on the levels of hepatic triacylglycerides, with a reduction of 20.6% relative to the drug alone. The association of protein-rosuvastatin produced an unexpected action on serum total cholesterol, maintaining it at the level shown by the HC group, which was 25 and 21% higher than those observed with the protein and drug alone. However, it also caused an increase in HDL-C of 23%, compared to protein and drug alone. Surprisingly, it achieved a reduction in the cardiovascular risk of 55%, relative to group HC.

## Competing interests

The authors declare that they have no competing interests.

## Authors' contributions

VAN and ESF designed the research; ESF and MAS conducted the research; ESF, VAN and AD analyzed the data; VAN and ESF wrote the paper; and VAN had primary responsibility for the final content. All authors read and approved the final manuscript.
